# A Review of Toxoplasmosis and Neosporosis in Water Buffalo (*Bubalus bubalis*)

**DOI:** 10.3389/fvets.2020.00455

**Published:** 2020-08-11

**Authors:** Luiz Daniel de Barros, João Luis Garcia, Katia Denise Saraiva Bresciani, Sérgio Tosi Cardim, Victor Sesnik Storte, Selwyn Arlington Headley

**Affiliations:** ^1^Laboratory of Animal Protozoology, Department of Preventive Veterinary Medicine, Universidade Estadual de Londrina, Londrina, Brazil; ^2^School of Veterinary Medicine Araçatuba, Universidade Estadual Paulista, Araçatuba, Brazil; ^3^Department of Veterinary Medicine, Universidade Norte Do Paraná, Arapongas, Brazil; ^4^Laboratory of Animal Pathology, Department of Preventive Veterinary Medicine, Universidade Estadual de Londrina, Londrina, Brazil

**Keywords:** *Toxoplasma gondii*, *Neospora caninum*, water buffalo, *Bubalus bubalis*, epidemiology

## Abstract

Toxoplasmosis and neosporosis are diseases with worldwide distribution that are associated with reproductive problems in livestock and responsible for economic losses. This review presents an overview of the current knowledge relative to these diseases in water buffalo (*Bubalus bubalis*). In general, buffalo are considered resistant to clinical toxoplasmosis because there are studies only reporting serological evidence of natural infection in these animals. Studies have described age, poor hygienic status of the farm, and presence of cats as risk factors for the development of *Toxoplasma gondii* infection in buffalo. It must be highlighted that buffalo meat, which does not receive adequate freezing treatment, could be a potential source for toxoplasmic human infection as well as the importance of raw buffalo milk in the transmission of toxoplasmosis to human beings. *Neospora caninum* is considered one of the major causes of abortion and responsible for huge economic losses in cattle. Vertical transmission is the main route to infect calves, and is responsible for maintaining the parasite within a herd. In buffalo, vertical transmission is also described; moreover, although there are indications that *N. caninum* may be associated with abortion in dairy buffalo, the reproductive importance of neosporosis is apparently lower in buffalo relative to cattle. Most studies have identified a higher time of exposition to *N. caninum* oocysts relative to age. The household system was also described as a risk factor for infection, possibly due to persistent contact between the home-raised buffalo and canids. The fetal immune competence of buffalo is similar to bovine, and buffalo fetus are highly susceptible to infection during the first trimester of pregnancy, indicating that *N. caninum* may be an abortigenic agent in buffaloes. Alternatively, it is interesting to note there is evidence that the inflammatory response in pregnant buffalo infected with *N. caninum* is mild enough to avoid abortion in most cases. It is proposed that the possible transmission of toxoplasmosis through unprocessed milk and buffalo meat may occur, which is important in terms of public health. Additionally, there is strong evidence to suggest that *N. caninum* may be associated with abortion in buffalo.

## Introduction

The domestic water buffalo (*Bubalus bubalis*) is originated from the domestication of the Indian wild buffalo (*Bubalus arnee*), which occurred ~5,000 years ago (1). Currently, there is an estimated population of 202 million heads, predominantly in Asia (97%), followed by Africa (1.7%) and South America (1%) (2). The buffalo population has been increasing in the last 10 years, probably because these animals are more resistant to infectious diseases and have better converting rates of poor-quality forage into milk and meat than cattle (1–3).

Toxoplasmosis and neosporosis are diseases caused by protozoan *Toxoplasma gondii* and *Neospora caninum*, respectively. These parasites have worldwide distribution and are associated with reproductive problems in livestock and responsible for economic losses (4–6). Buffalo appear to have fewer clinical problems with *T. gondii*, and only serological reports have published; however, it is a zoonotic disease, and attention should be taken relative to the drinking of buffalo milk. Regarding neosporosis in buffalo, although there are several serological studies, the importance of this disease as an abortive agent remains uncertain (7). Therefore, this review presents an overview of the current knowledge of toxoplasmosis and neosporosis in buffalo.

## Toxoplasmosis

### Introduction

*Toxoplasma gondii* is a protozoan parasite that is able to infect different animal species, including humans, and has a worldwide distribution (5). Felids are the definitive hosts, being able to shed unsporulated oocysts through feces, which, after sporulation in the environment, can infect many animals, and humans (5). Mammals and birds are the intermediate hosts due to the development of tissue cysts and can be infected by the ingestion of food and water contaminated with sporulated oocysts or by the ingestion of raw or undercooked meat contaminated with tissue cysts (8). Moreover, vertical transmission is an important form of parasitic infection in humans and domestic animals, such as pigs and sheep (9–11). The infection in immunocompetent humans is usually asymptomatic; however, recent studies have reported clinical cases associated with outbreaks (12, 13). In livestock, especially in goats and sheep, toxoplasmosis is responsible for economic losses, mainly due to reproductive disorders, which include abortion, stillbirth, and birth of weak animals (4). The economic impact of toxoplasmosis includes the costs associated with treatment and the reduction in the expected output production (14). In general, buffaloes are considered resistant to the clinical disease, and thus, there are studies only reporting serological evidence of natural infection in these animals.

### Epidemiology

**Figure 1:**
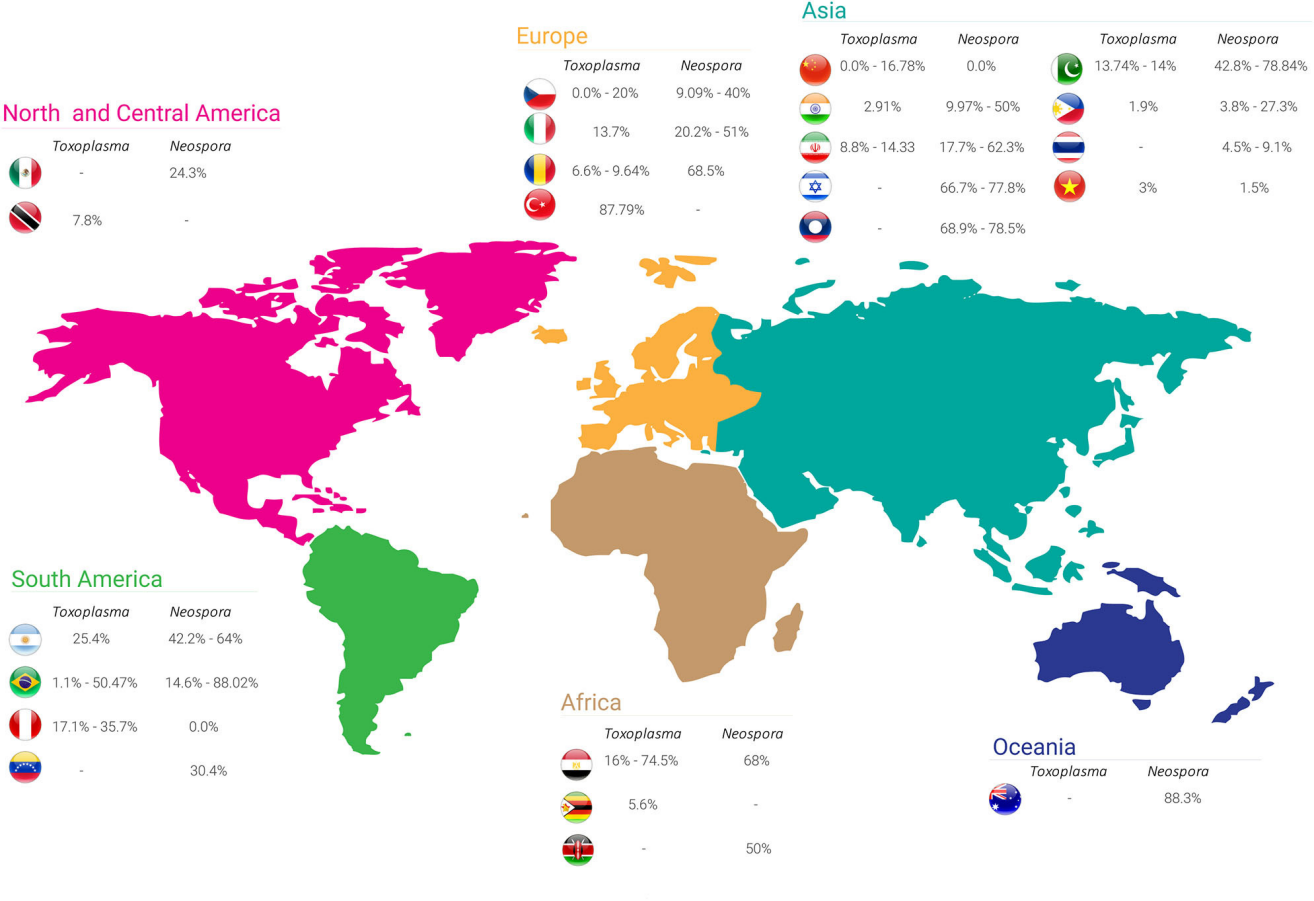
Seroprevalence rates of *Toxoplasma gondii* and *Neospora caninum* in buffalo worldwide.

Because buffalo meat and milk are used for human consumption, concern relative to the sanitary condition in buffalo herds has increased over the last 10 years (15). Several serological evaluations published worldwide have estimated that the serological status of the contact between buffaloes and *T. gondii* attained prevalence rates ranging from 0 to 87.79%. Table 1 shows the serological studies for *T. gondii* in buffalo worldwide, and Figure 1 summarizes the prevalence rates. Different serological techniques, mainly targeting IgG antibodies, for the diagnosis of toxoplasmosis in buffalo were used; these include the indirect fluorescent antibody test (IFAT), enzyme-linked immunosorbent assay (ELISA), modified agglutination test (MAT), Sabin-Feldman dye test (SFDT), latex agglutination test (LAT), and indirect hemagglutination (IHA). An ELISA assay was used to detect the IgM antibody for the diagnosing of acute infection in buffalo (39); however, the effectiveness of this assay is difficult to evaluate because this antibody is maintained only for few weeks after infection (48). The isolation of the parasite performed by mouse bioassay is the gold standard for the detection of *T. gondii* from tissue (5). A study evaluated the diaphragm from slaughter buffalo to isolate *T. gondii*; however, none of the infected mice were positive even those who were inoculated with tissue positive by PCR (46). A bioassay in cats was used to isolate *T. gondii* from the milk of buffalo, demonstrating that this model can be useful to isolate the parasite from different biological samples (49).

**Table 1:** Seropositivity of *Toxoplasma gondii* in buffalo from different countries.

**Location**							
**Continent**	**Country**	**Origin**	**Test**	**No. examined animals**	**% positive**	**Cutoff**	**References**
Africa	Egypt	Slaughterhouse	MAT^a^	75	16	25	(16)
	Egypt	Farm	ELISA^b^/LAT^c^	55	74.5/20	n.a.^i^/n.i.^j^	(17)
	Egypt	Slaughterhouse	MAT	160	22.5	25	(18)
	Zimbabwe	Wild (*Syncerus caffer*)	MAT	18	5.6	25	(19)
America	Argentina	Farm	IFAT^d^	500	25.4	100	(20)
	Brazil	Farm	IFAT	136	12.5	64	(21)
	Brazil	Farm	IFAT/ELISA	4796	34.9/40.32	40	(22)
	Brazil	Farm	IFAT	500	38.5	64	(23)
	Brazil	Farm	IFAT/ELISA	321	50.47/42.99	64	(24)
	Brazil	Farm	LAT	104	3.85	64	(25)
	Brazil	Farm	IFAT	169	27.2	64	(15)
	Brazil	Farm	IFAT	374	1.1	64	(26)
	Brazil	Farm	ELISA/IFAT	447	41.6/36.0	64	(27)
	Brazil	Slaughterhouse	IFAT	220	16.8	100	(28)
	Brazil	Farm	IFAT	411	49.9	64	(29)
	Peru	Farm	IHA^e^/IFAT	70	35.7/17.1	16/64	(30)
	Trinidad	Farm	LAT	333	7.8	64	(31)
Asia	China	Farm	IHA	83	0.0	64	(32)
	China	Farm	IAT^f^	40	0.0	n.i.	(33)
	China	Farm	IHA	427	7.5	64	(34)
	China	Farm	IHA	560	16.78	64	(35)
	India	Farm	ELISA	103	2.91	n.a.	(36)
	Iran	Farm	IFAT	385	8.8	16	(37)
	Iran	Slaughterhouse	MAT	300	14.33	25	(38)
	Pakistan	Farm	ELISA	422	13.74	n.a.	(39)
	Pakistan	Abattoir/Farm	LAT	50	14.0	64	(40)
	Philippines	Farm	ELISA	105	1.9	n.a.	(41)
	Vietnam	Abattoirs	DAT^g^	200	3	50	(42)
Europe	Czech and Slovak	Zoo (*Syncerus caffer, Syncerus caffer nanus, Bubalus arnee*)	IFAT	11	18.18	40	(43)
	Czech Republic	Captivity	IFAT/ELISA	5	20/0.0	50	(44)
	Italy	Farm	ELISA	124	13.7	n.a.	(45)
	Romania	Farm/Slaugtherhouse	MAT/ELISA	197	9.64/6.6	6	(46)
	Turkey	n.i.	SDFT^h^	131	87.79	16	(47)

a *Modified agglutination test*.

b *Enzyme-linked immunosorbent assay*.

c *Latex agglutination test*.

d *Indirect fluorescent antibody test*.

e *Indirect hemagglutination*.

f *Indirect agglutination test*.

g *Direct agglutination test*.

h *Sabin-Feldman dye test*.

i *not applied*.

j *not informed*.

Many studies have evaluated risk factors associated with *T. gondii* infection in buffalo (15, 31, 38, 39, 46, 47, 50). A significant difference was observed in the seroprevalence of buffalo from Mexico according to environmental conditions, where water buffalo raised in regions with annual rainfall between 1,266 and 1,650 mm had higher chances to be positive than those raised in regions with higher mean annual rainfall (50). Higher prevalence of IgM antibodies was verified in the monsoon season in Punjab, Pakistan, where the relative humidity, rainfall, and temperature are higher; these are ideal conditions for sporulation of oocysts in the environment (39). Changes in global climatic conditions have already been associated with a possible increase of *T. gondii* prevalence in humans from some regions of Europe (51).

Seroprevalence was considered to be comparatively more elevated in adult buffalo when compared with calves and juveniles (46). Other studies have also described age as a risk factor for the development of *T. gondii* infection in buffalo, where older animals are more seropositive than their younger counterparts (15, 31, 39), probably due to the higher chance of older animals being exposed to the parasite as age increases. Most studies have not observed a significant association between the gender and seropositivity for toxoplasmosis (31, 39, 46, 47, 50); however, a higher prevalence in male buffalo from Iran was observed, but all animals were under 2 years old, the age that was statically associated with infection (38). Conversely, a significant seroprevalence higher in female buffalo from Pakistan was verified with seropositivity being related to a lower immune response during the pregnancy and lactation periods (39).

When the type of rearing system was evaluated, a semi-intensive and extensive rearing system showed higher seropositive animals compared to the intensive system, probably associated with the greater risk of animals ingesting oocysts present in pastures and becoming infected (21, 39). Differently, studies done with buffalo from Romania and Trinidad did not observe any association between rearing systems and the occurrence of antibodies anti-*T. gondii* in buffalo (31, 46), maybe because of the climatic conditions in these countries.

Other risk factors associated with seroprevalence of toxoplasmosis in buffalo described in the literature are poor hygienic status of the farm and the presence of cats (39). Similar results were described in cattle, demonstrating the importance of cats in the epidemiology of *T. gondii*, because cats are usually common on farms and can shed millions of oocysts through feces, contaminating the environment (14, 52).

Reproductive disorders are usually associated with infection by *T. gondii*; however, there is only one study in water buffalo that analyzed this association, where two (0.6%) out of 307 female buffaloes from Mexico had a history of abortion and antibodies against *T. gondii*, resulting in a non-statistically significant association between toxoplasmosis and abortion in water buffalo (50). A recent study reported an association between *T. gondii* infection and a high number of days open (number of days from calving to conception) in water buffalo, and the authors hypothesized that the disease could result in embryonic death and resorption (45).

A study evaluated the presence of antibodies by MAT from 18 wild buffalo, including African buffalo (*Syncerus caffer*) from Zimbabwe, but only one animal was positive with titer of 25 (19). Five African buffalo (*Syncerus caffer caffer*), two Dwarf forest buffalo (*Syncerus caffer nanus*), and four water buffalo (*Bubalus arnee*) from a zoo in the Czech Republic were evaluated by IFAT, but only one African buffalo and one water buffalo were positive with titers of 80 and 640, respectively (43).

### Experimental Infections

To the best of the author's knowledge, there is only one study that performed an experimental infection of *T. gondii* in buffalo, and it used eight calves inoculated with a *T. gondii* GT1 strain, five at 10^5^ and the other three with 5 × 10^5^ oocysts (53). Five animals developed moderated pyrexia, anorexia, weakness, and dyspnea; one calf inoculated with the higher dose died 11 days post-inoculation (p.i.); and another inoculated with 10^5^ had to be euthanized 23 days p.i. because it was unable to eat and stand. Hematological changes were not observed, and the immunological response was first observed on day 21 p.i. by IHA at a cutoff of 64. The antibodies persisted for 63 days and then declined below the cutoff. By mouse bioassay, *T. gondii* was isolated from the brain, lungs, liver, kidney, lymph nodes, and spleen from the calf that died of acute toxoplasmosis. Samples from the lymph nodes and retina were used to isolate the parasite from the calves that were euthanized at 23 and 32 days p.i. These authors reported that the pathological analysis revealed mild interstitial pneumonia in one calf without histopathological evidence of tissue cysts in any of the histological sections (53). The results derived from this study suggest that buffalo, like cattle, are considered resistant to clinical toxoplasmosis and probably do not play an important role in the epidemiology of *T. gondii*.

### Public Health Concerns

Although different studies have described seropositivity in buffalo worldwide, the importance of buffalo meat for public health is currently unknown. In cattle, previous studies have not detected viable parasites in beef from meat stores (54); however, *T. gondii* should be considered an issue of public health in regions where buffalo meat is widely consumed (55). Fresh and imported frozen buffalo meat obtained from markets in Egypt were assessed for viable *T. gondii* by bioassay in cats, and the prevalence of tissue cysts in fresh meat was 15.4% although none of the frozen buffalo meat was considered positive (56). The process of freezing meat at −20°C during importation probably inactivates the tissue cysts because freezing meat to temperature of −12°C or below is efficient in destroying tissue cysts (5).

*Toxoplasma gondii* tissue cysts were isolated and detected by light microscopy of percoll dilutions in 15% of frozen buffalo meat that was illegally imported to Turkey from Iraq although all positive samples were confirmed by nested PCR using the B1 gene (57). Additionally, the meat was treated at −18°C for 2 days, and tissue cysts were not identified after this process. It must be highlighted that, based on these results, the meat from this study did not receive adequate freezing treatment and could be a potential source for human infection.

Raw milk samples from 33 buffalo herds from Iran were collected from clinically healthy animals and without alterations in physical characteristics of milk for the detection of *T. gondii* (49). Out of 164 milk samples obtained from buffalo, 4.26% were considered positive at Vero cell culture, and 3.65% and 3.04% were positive by capture ELISA and B1 gene PCR, respectively. Additionally, positive samples by the cell culture were used for cat bioassay; animals were fed with 50 ml of milk from buffalo for 3 days, and all challenged cats shed oocysts in the feces, demonstrating viable tachyzoites present in the milk (49). These results demonstrate the importance of raw milk in the transmission of toxoplasmosis to humans.

## Neosporosis

### Introduction

*Neospora caninum* is a protozoan parasite responsible for important diseases in dogs and cattle (58). In cattle, *N. caninum* is considered one of the major causes of abortion and responsible for severe economic losses (6). To date, only domestic dogs (*Canis familiaris*), Australian dingoes (*Canis lupus dingo*), coyotes (*Canis* latrans), and gray wolves (*Canis lupus*) are considered definitive hosts of the parasite (59–62). Although serological positivity against *N. caninum* was reported in several animal species, only a few are considered intermediate hosts, including some mammalian and bird species (58, 63). In humans, the presence of antibodies anti-*N. caninum* was described; however, the zoonotic potential remains unknown (64).

Vertical transmission is the main route of infection in calves and is responsible for maintaining the parasite within a herd (65, 66). In buffalo, vertical transmission was also described, but the importance of this form of transmission in the epidemiology of the disease in buffalo remains to be elucidated. Moreover, the reproductive importance of neosporosis in buffalo has to be clarified; however, this importance is apparently lower than in cattle (7).

### Epidemiology

Table 2 shows the seroprevalence of neosporosis in buffalo worldwide with seropositivity ranging from 0 to 88.3% based on different serological assays. A summary of the seroprevalence rates of *N. caninum* in each country is shown in Figure 1. Although the seroprevalence in buffalo appears to be three times higher than in cattle, the association between *N. caninum* and reproductive disorders in buffalo seems not to be a common occurrence (6, 7).

**Table 2:** Seropositivity of *Neospora caninum* in buffalo from different countries.

**Location**							
**Continent**	**Country**	**Origin**	**Test**	**No. examined animals**	**% Positive**	**Cutoff**	**References**
Africa	Egypt	Slaughterhouse	NAT^a^	75	68	20	(16)
	Kenya	Game ranch (*Syncerus caffer*)	NAT	4	50	40	(67)
America	Argentina	Farm	IFAT^b^	449	64	100	(68)
	Argentina	Farm	IFAT	500	42.2	100	(20)
	Argentina	Farm	IFAT	1,350	43.3	100	(69)
	Brazil	Farm	IFAT	136	19.1	200	(21)
	Brazil	Farm	IFAT/ELISA^c^	47,96	48.8/55.55	100	(22)
	Brazil	Farm	IFAT	500	39	128	(23)
	Brazil	Farm	IFAT	345	35.4	100	(70)
	Brazil	Farm	IFAT/NAT	222	64/53	25/40	(71)
	Brazil	Farm	IFAT	196	70.9	25	(72)
	Brazil	Slaughterhouse	IFAT	220	36.4	100	(28)
	Brazil	Farm	IFAT	374	40.9	200	(26)
	Brazil	Farm	IFAT/ELISA	288	53.12/17.36	200	(73)
	Brazil	Farm	ELISA	164	14.6	n.a.^f^	(74)
	Brazil	Farm	ELISA	192	88.02	n.a.	(75)
	Brazil	Farm	IFAT	411	56.0	200	(29)
	Mexico	Farm	ELISA	144	24.3	n.a.	(76)
	Peru	Farm	IFAT/ELISA	83	0.0	100	(77)
	Venezuela	Farm	ELISA	174	30.4	n.a.	(78)
Asia	China	Farm	ELISA	40	0.0	n.a.	(33)
	India	Farm	cELISA^d^	32	50	n.a.	(79)
	India	Farm	cELISA	341	9.97	n.a.	(80)
	India	Farm	cELISA	51	21.6	n.a.	(81)
	Iran	Slaughterhouse	ELISA	181	37	n.a.	(82)
	Iran	Farm	ELISA	122	62.3	n.a.	(83)
	Iran	Abattoir	ELISA	83	19.27	n.a.	(84)
	Iran	Farm	ELISA	236	17.7	n.a.	(85)
	Israel	n.i.^g^	MAT/IFAT/WB^e^	18	77.8/72.2/66.7	200/200/20	(86)
	Laos	Farm	ELISA	130	78.5	n.a.	(87)
	Laos	Farm	ELISA	61	68.9	n.a.	(88)
	Pakistan	Farm	cELISA	300	54.7	n.a.	(89)
	Pakistan	Farm	cELISA	312	42.8	n.a.	(90)
	Pakistan	Farm	IFAT	52	78.84	200	(91)
	Philippines	Farm	ELISA	105	3.8	n.a.	(41)
	Philippines	Farm	cELISA	176	27.3	n.a.	(92)
	Thailand	Farm	IFAT	628	9.1	100	(93)
	Thailand	Farm	ELISA	532	4.5	n.a.	(94)
	Vietnam	Farm	IFAT/ELISA	200	1.5	640	(42)
Europe	Czech and Slovak	Zoo (*Syncerus caffer, Syncerus caffer nanus, Bubalus arnee*)	IFAT	11	9.09	40	(43)
	Czech Republic	Captivity	IFAT/cELISA	5	40.0/20.0	50	(44)
	Italy	Farm	IFAT	1,377	34.6	200	(95)
	Italy	Farm	ELISA	908	51.0	n.a.	(96)
	Italy	Farm	ELISA	124	20.2	n.a.	(45)
	Romania	Farm/Slaughterhouse	ELISA	197	68.5	n.a.	(97)
Oceania	Australia	Farm	ELISA	480	88.3	n.a.	(98)

a *Neospora caninum agglutination test*.

b *Indirect fluorescent antibody test*.

c *Enzyme-linked immunosorbent assay*.

d *Competitive-inhibition enzyme-linked immunosorbent assay (cELISA)*.

e *Western blot*.

f *not applied*.

g *not informed*.

Indirect ELISA and IFAT were the main techniques employed for the serological diagnosis of neosporosis in buffalo; however, a competitive-inhibition ELISA (cELISA) and a *Neospora* agglutination test (NAT) were also used. The NAT assay is similar to the MAT assay for the identification of toxoplasmosis; however, it uses *N. caninum* tachyzoites. Moreover, this technique can be done in different animal species because it does not require an anti-IgG conjugate and shows high sensitivity and specificity (99). An indirect ELISA was developed based on fragments of recombinant NcGRA7 for the diagnosis of *N. caninum* infection in buffalo (83). This new ELISA demonstrated 98.6% sensitivity and 86.5% specificity using buffalo sera, obtained a good correlation with a commercial ELISA, and seems to be a good tool for the serological screening of neosporosis in buffalo.

The presence of an immunodominant 17–18 kDa antigen by Western blot was used to confirm the presence of antibodies anti-*N. caninum* in buffalo from Israel (86). Western blot confirmed infection in 85.7% (12/14) of the positive samples by MAT and 84.6% (11/13) by IFAT, demonstrating high sensitivity for both techniques (86). Recently, a milk ELISA was evaluated as an alternative method to detect *N. caninum* antibodies in lactating buffalo (91). The milk iscom ELISA detected a comparatively reduced number of positive milk samples than competitive ELISA using serum samples; however, a moderate agreement between the techniques was observed, indicating that this milk ELISA could be used in lactating dairy herds with a high prevalence of neosporosis (91).

Bioassay in dogs, gerbils, and cell culture were done to isolate *N. caninum* from brain tissues of six buffalo seropositive by IFAT (cutoff = 100) (100). Four dogs shed oocysts through feces after being fed buffalo brain, the higher number of oocysts shed was 820,655, and the higher duration of oocyst shedding was 26 days. These authors report that, by cell culture, *N. caninum* was isolated from the brains of two buffalo, and via bioassay in gerbils, the parasite was isolated from the brains of three buffalo. Additionally, the isolates, named NCBrBuf-1 to NCBrBuf4, were confirmed as *N. caninum* by PCR and DNA sequencing, targeting the Nc5 and ITS-1 region, and showed a low degree of genetic diversity between other *N. caninum* isolates (100). Collectively, these results demonstrate that buffalo are natural intermediate hosts for *N. caninum*; this study is the first report of isolation of the parasite in this species (100).

A recent study revealed a higher seroprevalence for *N. caninum* in adults relative to calves and young buffalo (97). This result is in accordance with previous studies (20, 68, 69, 82, 87, 95) and may be related to a comparative higher period of exposition to oocysts relative to age. A higher prevalence in older buffalo (more than 10 years old) was verified, suggesting that horizontal transmission occurs more commonly in buffalo than vertical transmission (93). Another study demonstrated an association between age and *N. caninum* seroprevalence by IFAT with higher prevalence in animals that were 6–11 years old; however, this association was not observed when these samples are analyzed by NAT (71). Alternatively, previous studies from Brazil (72), India (80), and Thailand (94) did not demonstrate statistical differences among age-related groups and neosporosis. When the gender of buffalo was compared, differences in infection by *N. caninum* were not identified between male and female in studies from Argentina (68), Thailand (93), and Pakistan (89, 90); however, serological surveys done in Iran (82) and Romania (97) showed statistical differences for gender with higher prevalence in female buffalo.

In female water buffalo from Brazil, the risk factors associated with neosporosis were grazing and animals derived from animal markets and/or reputable sellers (70), reinforcing the hypothesis of postanal transmission due to the ingestion of food or water contaminated with oocysts. By logistic regression analysis, the presence of pigs was considered as a risk factor for neosporosis in buffalo in Paraíba state, Brazil (21), although pigs do not play an important role in the epidemiology of *N. caninum*. The household system was also described as a risk factor by *N. caninum*, probably due to the persistent contact between home-raised buffalo and canids (97).

Contact with dogs was associated with infection in buffalo from Pakistan, suggesting the role of dogs in contaminating buffalo feed, resulting in horizontal transmission (89). In this same study, summer weather was also associated with seroprevalence of *N. caninum* in buffalo, and it was suggested to be related to the gestational phase of these animals because, during the Pakistan summer, most animals are in midgestation, which could result in changes in the immune system and parasite transmission (89).

Although reproductive disorders, such as abortion, appear to be less common in buffalo relative to cattle, the prevalence of *N. caninum* antibodies was significantly higher in dairy buffalo that had a history of abortion than those without any abortive history, indicating that this agent may be associated with abortion in dairy buffalo (89). It was reported that, in 167 buffalo seropositive for *N. caninum*, 13.2% (22/167) were co-infected with *Brucella abortus*, indicating a higher abortion risk in these animals than if infected with only one of these pathogens (90). Similarly, *N. caninum* infection was associated with abortion and retained fetal membranes, indicating that neosporosis can be related to reproductive disorders in water buffalo (45).

*Neospora*-like cysts were observed in the brain of the fetuses of two buffalo by histological analysis on a farm in Italy that had a history of abortion and death of buffalo calves with neurological manifestations (95); however, additional confirmation of the identity of the intralesional cysts by other diagnostic methods, such as immunohistochemistry on *in situ* hybridization, was not done to confirm these tissue cysts as *N. caninum* (95). It was demonstrated that, in 17 calves born from seropositive dams, antibodies anti-*N. caninum* persisted for 12 months, suggesting neonatal transmission of the parasite in buffalo, probably by the transplacental route (101). However, the first evidence of congenital transmission of *N. caninum* in buffalo was suggested in a 3-month-old fetus that was negative at IFAT and brain tissue positive by a nested PCR assay that targeted the ITS1 and Nc5 regions (102). These conflicting results could be due to the immunocompetency of the fetus (102).

DNA from *N. caninum* was detected by qPCR in the fetus of one water buffalo from a fetus from a dam that was 2–3 years old, during which abortion occurred between 180 and 210 days of gestation (103); however, the authors concluded that they could not have confirmed if neosporosis was the only cause of abortion because other abortive agents could be present. Consequently, they suggest that *N. caninum* could be responsible for reproductive failure in water buffalo. *N. caninum* DNA was identified by 18S PCR assay in the brains and hearts of three buffalo fetuses that suffered spontaneous abortion between the fourth and 6 months of gestation (96). Additionally, these fetal tissues were considered negative for the major pathogens responsible for reproductive failures in buffalo, including *Brucella* spp., *Salmonella* spp., *Chlamydophila* spp., *Listeria* spp., *Campylobacter* spp., *Coxiella burnetti, Leptospira* spp., *Toxoplasma gondii*, Bovine and Bubaline Herpesvirus (BoHV1 and BuHV1), and bovine viral diarrhea virus (BVDV), indicating *N. caninum* as an important abortive agent (96). In a recently published study, a *Neospora-*associated abortion was reported for the first time in buffalo from India, where tissues from two fetuses were positive by immunohistochemistry (81).

The presence of the parasite DNA was detected by nested PCR assay only at the diaphragm (heart and mesenteric lymph node were also evaluated) from six animals (8.1%, 6/74) of different ages; three of these animals also were negative by serological assays (97). These results demonstrate that the diaphragm may be an adequate tissue for the detection of *N. caninum* in buffalo and that serologically negative animals can also have the parasite in their tissues.

Recently, it was demonstrated that buffalo with higher titers of antibodies against *N. caninum* also contained parasite DNA in the serum samples, being the first report to confirm *N. caninum* DNA in serum samples of buffalo and indicating that parasitemia occurs simultaneously with antibody production (84).

### Experimental Infections

The first experimental study with *N. caninum* in buffalo was performed in 2005 when the authors inoculated six buffalo with 5 × 10^6^ tachyzoites of Illinois strain by subcutaneous route (101). All animals were positive between 7 and 11 days p.i. by IFAT with a cutoff of 25, and titers peaked at 3 weeks p.i. and remained elevated until 7 weeks p.i., after which the peak started to decrease (101).

Intravenous inoculation of 10^8^ tachyzoites of the NC-1 strain was performed in an experimental study involving 10 female water buffalo at 70 (*n* = 3) or 90 (*n* = 7) days of pregnancy (104). Although all dams developed specific antibodies with titers ranging from 3,200 to 51,200, none of these showed clinical signs or aborted; however, one non-viable fetus was observed from a female inoculated at 70 days of gestation and slaughtered at 28 days p.i. (104). Specific antibodies against *N. caninum* (cutoff = 10) were detected in only two fetuses; both dams were inoculated at 90 days of gestation and slaughtered at 72 days p.i. Moreover, *N. caninum* DNA was detected by nPCR in the placenta and fetuses of all inoculated animals (104). These results indicate that fetal immune competence of buffalo is similar to that of cattle at ~110 days of gestation, and fetuses of buffalo are highly susceptible to infection during the first trimester of pregnancy, indicating that *N. caninum* should be considered as an abortigenic agent in water buffalo.

An experimental infection was done to evaluate the outcome of infection with different strains of *N. caninum* in six pregnant buffalo at the 70th day of gestation with 5 × 10^8^ tachyzoites by intravenous route (105). Three pregnant buffalo with the NC-1 strain and the others with the NC-Bahia, a Brazilian strain isolated from the brain of a naturally infected dog. Additionally, all the females developed antibodies detected by ELISA and IFAT (cutoff = 50), but none of the fetuses were considered seropositive (cutoff = 25). Moreover, fetal death occurred in all females infected with the NC-1 strain but not in dams inoculated with the NC-Bahia strain. Furthermore, a semi-nested PCR detected *N. caninum* DNA in maternal and fetal tissues, indicating transplacental transmission in all animals, but the NC-Bahia seems to be less virulent for pregnant buffalo (105).

### Pathogenesis

*Neospora caninum* is recognized as one of the major causes of reproductive failures in cattle; however, little is known about the pathogenesis of the disease in buffalo. So far, natural abortion in buffalo by neosporosis was only reported in one study (96), and all knowledge relative to the pathogenesis is based on experimental observations.

Non-suppurative encephalitis and myocarditis were observed in aborted fetuses, and few *N. caninum–*like cysts were identified in histologic sections of the brain, but none of the molecular techniques were used, compromising the diagnoses as *N. caninum* (95).

Challenged pregnant female buffalo with *N. caninum* tachyzoites developed non-suppurative placentitis as the more frequent histopathologic finding, being observed in 9 out of 10 specimens (104). Additionally, meningoencephalitis, characterized by gliosis and non-suppurative inflammatory infiltration at the pia mater with multiple necrotizing foci at the central nervous system, were observed in fetuses derived these female buffalo. Moreover, in other fetal tissues, including the lungs, heart, kidneys, striated muscle, and liver, multiple foci of mononuclear inflammatory cells were observed in almost all samples analyzed, and immunohistochemistry identified intralesional tachyzoites in two fetuses that were associated with focal necrosis and gliosis in the central nervous system in one of these (104).

Non-suppurative inflammation in placentomes and fetal tissue was also observed after infection of nine pregnant buffalo in early gestation with the NC-1 strain of *N. caninum* (106). Moreover, a mononuclear inflammatory infiltrate was identified surrounding the necrotic foci at the caruncle or within necrotic fetal villi in the placentome of all dams challenged at 70 days of gestation with 10^8^ tachyzoites of the NC-1 strain of *N. caninum* and culled at 28 days p.i. with higher scores in dams carrying non-viable fetuses. These authors suggested that the inflammatory response in pregnant buffalo infected with *N. caninum* was mild enough to avoid abortion in most of the cases (106).

Lymphocytic infiltration was the main lesion observed in placental and fetal tissues after pregnant buffalo were inoculated with two strains of *N caninum* (105). Hemorrhagic hepatitis and nephritis were observed in the fetuses, and necrosis at the placenta and fetal brain were also observed. These authors indicate that the fetuses from the dam inoculated with the NC-Bahia strain contained fewer lesions with comparatively less severity when compared with fetuses inoculated with NC-1 strain, indicating that the NC-Bahia strain is more pathogenic than the NC-1 strain of *N. caninum* (105).

Mild satellitosis of the brain and perivascular macrophage at the heart were observed in two pregnant buffalo inoculated with NC-1 tachyzoites (75). Additionally, periportal non-suppurative hepatitis with centrilobular infiltration of macrophages and perivascular mononuclear cell infiltration at the kidney was also observed in these females. Moreover, brain tissues from fetuses from these pregnant buffalo presented multifocal necrosis and gliosis associated with infection by *N. caninum*, and the fetuses revealed non-suppurative myocarditis and pericarditis with macrophage infiltration, myositis, lymphocytic and macrophagic portal hepatitis, and a non-suppurative nephritis with perivascular and periglomerular lymphocytic and macrophage infiltration. Furthermore, the kidney of one of the fetuses had hemorrhage in the renal pelvis, and lung tissue from other fetuses showed peribronchiolar lymphocytic and macrophage infiltration with multifocal hemorrhage (75).

## Conclusions

The buffalo industry is growing worldwide, and the review of the literature indicates that the possible transmission of toxoplasmosis through either unprocessed milk or buffalo meat may occur. Furthermore, considering the high *T. gondii* seroprevalence observed in these animals, we could consider that the public health risk incurred by persons exposed to infected animals is probably higher than previously assumed. We summarized all seroprevalence studies of *N. caninum* in buffalo, which showed a high prevalence worldwide Additionally, there is strong evidence to suggest that *N. caninum* may be associated with abortion in buffalo. Finally, the present review will contribute to lightening future studies in this field.

## Author Contributions

LB, JG, KB, SC, VS, and SH contributed to conception, design, drafting, and critical revision of the manuscript. All authors contributed to the article and approved the submitted version.

## Conflict of Interest

The authors declare that the research was conducted in the absence of any commercial or financial relationships that could be construed as a potential conflict of interest.
